# Determining the Precise Cerebral Response to Acupuncture: An Improved fMRI Study

**DOI:** 10.1371/journal.pone.0049154

**Published:** 2012-11-09

**Authors:** Hua Liu, Jianyang Xu, Baoci Shan, Yongzhong Li, Lin Li, Jingquan Xue, Binbin Nie

**Affiliations:** 1 Institute of High Energy Physics, Chinese Academy of Sciences (CAS), Beijing, China; 2 Graduate University of Chinese Academy of Sciences, Beijing, China; 3 Key Laboratory of Nuclear Analysis Techniques (LNAT), CAS, Beijing, China; 4 General Hospital of Armed Police Forces, Beijing, China; 5 Xuanwu Hospital, Capital Medical University, Beijing, China; University of Maryland, United States of America

## Abstract

**Background:**

In acupuncture brain imaging trials, there are many non-acupuncture factors confounding the neuronal mapping. The modality of the placebo, subjects’ psychological attitude to acupuncture and their physical state are the three most confounding factors.

**Objective:**

To obtain more precise and accurate cerebral fMRI mapping of acupuncture.

**Design and Setting:**

A 2×2 randomized, controlled, participant-blinded cross-over factorial acupuncture trial was conducted at Xuanwu Hospital in Beijing, China.

**Participants:**

Forty-one college students with myopia were recruited to participate in our study and were allocated randomly to four groups, Group A, Group B, Group C and Group D.

**Interventions:**

Group A received real acupuncture (RA) and treatment instruction (TI); Group B received RA and non-treatment instruction (NI); Group C received sham acupuncture (SA) and TI; Group D received SA and NI.

**Results:**

Stimulation at LR3 activated some areas of the visual cortex, and the cerebral response to non-acupuncture factors was complex and occurred in multiple areas.

**Conclusions:**

The results provide more evidence regarding the credibility of acupuncture therapy and suggest that more precise experimental designs are needed to eliminate sources of bias in acupuncture controlled trials and to obtain sound results.

## Introduction

Many studies have been performed using functional magnetic resonance imaging (fMRI) to investigate the cerebral matrix related to acupuncture therapy. [1], [2], [3], [4], [5] A rather variable pattern in blood oxygenation-level dependent (BOLD) signal changes were obtained in those studies, even at specific acupoints. These variable patterns are indicative of non-acupuncture factors that confound the results.

The creditability of the placebo is an important factor in acupuncture trials. [6] The most commonly used control modality is ‘sham acupuncture’, of which the depth of insertion and stimulation is the same as the RA, but the insertion points differ. During the procedure, sham acupuncture is matched as closely as possible with the real acupuncture. However, studies have pointed out that sham acupuncture appears to have an analgesic effect similar to that of real acupuncture. [7] Evidence from clinical trials suggests that sham acupuncture has little effect on nausea and is primarily a placebo. [6], [8].

Patients participate in some studies, whereas healthy volunteers are involved in others. LI4 (Hegu) is one of the most common acupoints to be investigated in analgesic studies. The consistency of the experimental results is, however, not ideal. [2], [9], [10] For a specific acupoint, the physical status of a subject should be taken into greater consideration.

Psychological factors, especially an individual’s expectations, can alter their physical condition and have treatment effects. [11] The same situation exists in acupuncture trials. [12] In acupuncture imaging trials, neither positive nor negative psychological tendencies should be eliminated.

In this fMRI study, we employed a 2×2 single-blind randomized cross-over factorial design to filter the cerebral responses from those non-acupuncture factors mentioned above and to obtain a more precise estimation of neuronal responses to acupuncture at LR3 (Taichong).

**Table 1:** Comparisons of behavioral results.

		RA		SA	
		Group A	Group B	Group C	Group D
Credibility scores comparison		QB QA		QB QA	
*P* value (*χ^2^*)	Group A	–	–	0.42 0.56	–
Needling sensation scores	Mean (SD)	14.25 (7.44)	12.46 (7.02)	4.23 (5.01)	3.77 (3.58)
comparison	95% CI	8.96–18.35	6.45–16.91	2.57–7.38	2.09–6.88
	Mean (SD)	13.75 (7.23)		3.97 (4.35)	
	95% CI	6.45–18.35		2.57–7.38	

Scores of credibility and belief comparison between Group A and Group C, and needle sensation for all four interventions.

RA: real acupuncture; SA: sham acupuncture; QB: questions before treatment (How confident are you that this treatment can alleviate your complaint?); QA: questions after treatment (How confident would you be in recommending this treatment to a friend who suffered from the same complaint?).

## Participants and Methods

### 1. Subjects

The study was approved by the Ethics Committee of the Chinese Academy of Sciences (Beijing, China). We obtained written informed consent from all participants involved in the study.

**Table 2:** Spatial coordinates and levels of significance of the activations.

Contrasts	Anatomical structure	Side	Talairach space coordinates			Statistical significance
			x	y	z	Z
RA−SA>0	VII	R	4	−83	32	2.91
	Visual-parietal area	R	35	−54	23	2.49
EG−NE>0	MFG	L	−2	35	48	4.06
	OFC	R	44	19	−13	2.85
		L	−42	25	−15	3.27
	DLPFC	R	42	38	26	2.96
		L	−24	61	8	3.07
	SII	R	67	−7	13	2.85
		L	−61	−23	12	2.73
	Temporal pole	L	−24	4	−30	2.84
	VII	R	36	−93	0	3.16
	Cerebellum	R	4	−71	−13	2.62
		L	−18	−85	−19	2.71
	Uncus	L	−24	4	−30	2.7
Group A−Group C>0	VII	R	6	−80	32	3.04
	Cerebellum	R	18	−30	−15	4.04
		L	−34	−45	−40	2.64
Group B−Group D>0	VII	L	−14	−59	18	2.79
	OFC	R	26	38	−19	3.72
Group A−GroupB>0	MFG	L	−1	33	35	3.09
	DLPFC	R	40	36	28	2.47
		L	−26	59	10	3.59
	OFC	R	46	19	−13	2.94
	SI/MI	L	−6	−39	65	2.55
		R	4	16	43	3.21
	SII	R	50	−52	50	2.79
		R	28	−65	60	3.42
	Temporal pole	L	−44	20	−23	2.88
	VI	R	20	−82	−11	3.57
	VII	R	28	−64	3	2.94
	Uncus	L	−24	2	−32	2.78
	Cerebellum	R	12	−59	−9	2.86
		L	−20	−85	−19	2.61
Group C−Group D>0	MFG	L	−4	37	50	3.36

p<0.001 uncorrected and k = 10 continuous voxels.

VII: secondary visual cortex; MFG: medial frontal gyrus; OFC: orbital frontal cortex; DLPFC: dorsum lateral prefrontal cortex; SI/MI: first sensory-motor cortex; SII: secondary sensory cortex.

Forty-one college students with myopia (male/female = 21/20, age 25.7±3.7 years, all right-handed) were recruited to participate in our study. No subjects had a history of neurological or psychiatric illness and all were acupuncture naive. Subjects were free of any transient medical problems and did not take any medications the day prior to the study.

**Figure 1:**
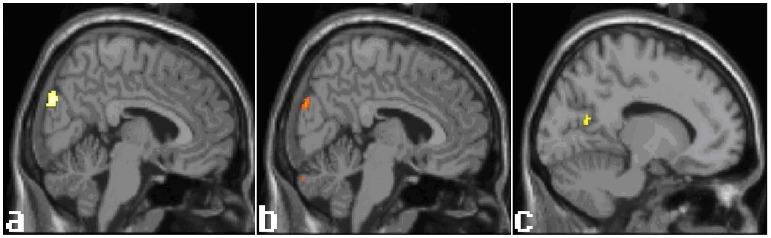
Activation of visual-related cortex by acupuncture. a: Activation in RA vs. SA. b: Activation in Group A vs. Group C. c: Activation in Group B vs. Group D. In both RA vs. SA and Group A vs. Group C, the activation occurred in the same right superior part of VII. The activation in Group B vs. Group D is in the interior part of the left VII. SPM maps the threshold at p<0.001, uncorrected for multiple comparison. See Table 2 for details. RA: real acupuncture (Group A with Group B). SA: sham acupuncture (Group C with Group D).

### 2. Experimental Groups and Acupuncture

All the subjects were divided into four groups randomly.

Subjects in Group A (n = 11) underwent real acupuncture (RA), and were told they would receive acupuncture treatment which would surely benefit their myopia before MRI scan (treatment instruction).

**Figure 2:**
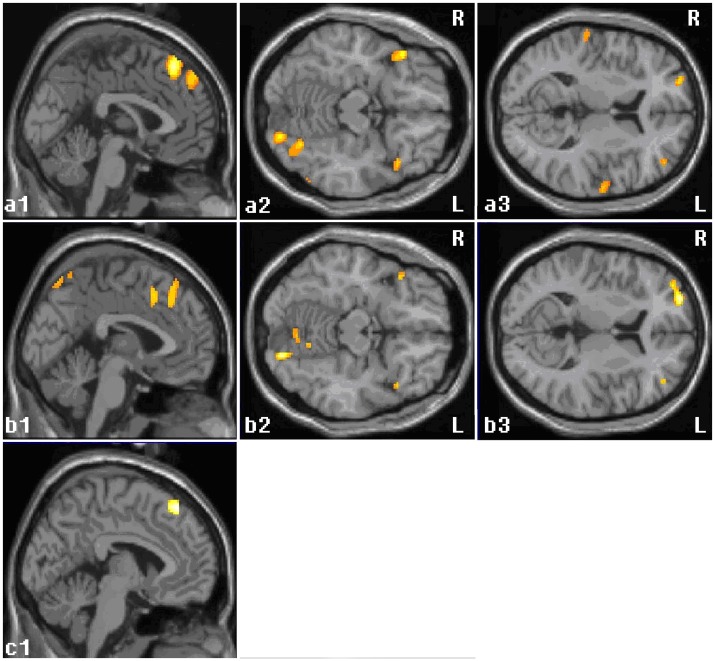
Activations related to the effect of expectancy interventions. a: Activations in EG vs. NE. b: Activations in Group A vs. Group B. c: Activations in Group C vs. Group D. In those three interventions, the left medial cortex, bilateral OFC and right posterior lobe of the cerebellum were activated (a1, a2, b1, b2, c1). In both EG vs. NE and Group A vs. Group B, bilateral DLPFC were activated (a3, b3). SPM maps the threshold at p<0.001 uncorrected. See Table 2 for details. EG: expectancy group (Group A with Group C). NE: no-expectancy group (Group B with Group D).

Subjects in Group B (n = 11) received RA stimulation, but were told they were participating in an experiment to estimate a medical machine’s sensitivity of detecting tactile signals (non-treatment instruction).

Subjects in Group C (n = 10) received stimulation at the sham acupoint (SA) and treatment instruction.

Subjects in Group D (n = 9) received stimulation at the SA and non-treatment instruction.

Each subject underwent only one of the four interventions, and each participant received the individual instructions only before the MRI scan. Each subject participated in the trial individually to avoid inter-participant interaction.

The acupuncture point left LR3 (Taichong) is located in the muscle bulk between the 1st and 2nd phalangeals on the dorsum of the left foot. One sham acupuncture point was chosen approximately 10 mm anterior to the classical site.

For Group A and Group C, before scanning, participants were asked two questions regarding credibility rating. [13] Each question was measured on a 0–6 Likert scale with 6 being the most credible.

All subjects completed a needle sensation questionnaire (NSQ) after scanning. We adopted an NSQ scale system to evaluate the needling sensation during acupuncture, with scores ranging from 0 (minimum) to 75 (maximum). [14].

After entering the scanning room, all subjects underwent resting scanning for 186 seconds (62 scans) first. A sterile, single-use silver needle (25 mm in length ×0.30 mm in diameter) was then inserted into the real acupoint or the sham acupoint, and the needle was twirled manually clockwise and counterclockwise at 1 Hz with even reinforcing and reducing manipulation for 180 seconds (60 scans). The needle was subsequently extracted while fMRI scanning continued for a total of 402 scans for every subject. The depth of needle insertion was approximately 10 mm, and all the acupuncture manipulations were performed by the same acupuncturist (Dr. Jianyang Xu, the General Hospital of the Armed Police Forces).

### 3. fMRI Procedure and Data Analysis

The experiments were performed with a 1.5-T whole body MRI scanner (Siemens, Sonata, Germany), with a standard head coil. Images spanned the entire head and were parallel to the anterior commissure-posterior commissure line. Functional images were obtained using a BOLD T2*-weighted gradient-echo EPI sequence with an in-plane resolution of 3.59 mm (TR = 3000 ms, TE = 50 ms, flip angle 90°, field of view = 230 mm×230 mm, matrix = 64×64 mm, 6-mm slice thickness and 1.2-mm slice gap).

The data were analyzed with statistical parametric mapping (SPM2) software (Welcome Department of Imaging Neuroscience, London, UK). The first two scans were discarded, so every subject had a final total of 400 volumes. After realignment, the images were normalized to the Montreal Neurological Institute (MNI) space and then smoothed spatially using a 9 mm×9 mm×9 mm Gaussian kernel. The estimated data were analyzed at two levels. The first level, for each individual subject, was a fixed-effect analysis based on the general linear model with a box-car response function as the reference waveform convolved with the Poisson hemodynamic response function. The cerebral areas activated during acupuncture relative to the baseline were obtained. The second level was performed using random-effect analysis based on a one-way analysis of variance model with fixed effect results to obtain intergroup comparisons. The results were reported using Talairach space coordinates.

## Results

### 1. Behavioral Results

#### Credibility

Credibility ratings were compared using a *χ^2^* test (Table 1). There were no statistical differences between scores for Group A and Group C.

#### Needle sensation

The mean scores for needle sensation are shown in Table 1. There are no statistical differences between Group A and Group B or between Group C and Group D. RA did elicit significantly different sensations from SA.

### 2. fMRI Results

SPM maps of the random effect analysis threshold were at p<0.001 uncorrected and k = 10 continuous voxels. Subjects receiving real acupuncture made up the RA group, and those receiving the sham acupuncture made up the SA group. Subjects receiving treatment instruction made up the EG group, and those not receiving treatment instruction made up the NE group. The comparison of spatial coordinates of area activations are listed in Table 2.

#### RA vs. SA

Activations occurred in the upper division of the right occipital cuneus, the secondary visual cortex (VII) (Fig. 1a).

#### EG vs. NE

Areas in the left medial frontal gyrus (MFG) (Fig. 2 a1), bilateral dorsum lateral prefrontal cortex (DLPFC) (Fig. 2 a3), bilateral orbital frontal cortex (OFC) (Fig. 2 a2) and bilateral posterior lobe of the cerebellum were activated.

#### Group A vs. Group C

The right superior division of the cuneus, part of VII (Fig. 1b), the right culmen of the cerebellar anterior lobe and the left cerebellar tonsil were activated.

#### Group B vs. Group D

The left interior division of VII (Fig. 1c) and the right OFC were activated.

#### Group A vs. Group B

The left medial frontal cortex (Fig. 2 b1), right OFC (Fig. 2 b2), SII, the visual-temporal area, the inferior division of VII, the anterior lobe of the cerebellum, and the bilateral DLPFC (Fig. 2 b3) were activated.

#### Group C vs. Group D

Areas in the left MFG (Fig. 2 c1) were activated.

## Discussion

The behavioral effect of acupuncture stimulation at the real acupoint is different from the effect at the sham acupoint. Furthermore, subjects receiving the treatment instruction exhibited sound expectancy due to the psychological effect of our instruction.

### 1. The Specific Effect of Acupuncture at LR3

According to Chinese traditional medicine, LR3 is a fundamental acupoint, and acupuncture at this point is used to treat many disorders including eye diseases. Some studies have found that acupuncture at LR3 decreases intraocular pressure. [15], [16] Those studies suggested that acupuncture at visual-related acupoints could modulate optical physiology. In this study, the superior part of the secondary visual cortex (VII) was activated in two contrasting conditions (RA vs. SA and Group A vs. Group C), and the interior part of VII was activated in the other contrasting condition (Group B vs. Group D). VII plays an important role in the integration of visual information and visual physiology. [17] How stimulation at certain point(s) modulates certain organs is the secret of acupuncture. In many trials examining the anesthesia effect of acupuncture, some pain-related regions are mentioned. [2], [18] Acupuncture may modulate central neural activity through a crossed spino-thalamo-cortico-limibic pathway or a direct uncrossed spino-thalmo-limibic pathway. [12], [19], [20] Acupuncture at LR3, an acupoint on the foot, may control eye-related cortex via those pathways; however, those studies did not give direct proof.

### 2. The Effect of Non-acupuncture

In this study, we adopted a “sham acupoint” as a placebo rather than other placebo modalities, such as minimal acupuncture, mock transcutaneous nerve stimulation (TENS) and Streitberger needle (SN). [21] TENS and SN can give subjects the impression of being pierced by an acupuncture, which are credible as placebos for psychological effects, but have no actually straight physiological effects. It means that the physical proprioception to needle stimulation is absent. We considered the “sham acupoint” as a placebo was credible and primarily acted as a placebo in this non-painful study.

The choice of subjects with myopia was based on the following consideration. Myopia is one relatively simple pathological condition and other systemic states are normal, so the complex response originating from irrelevant systems could be avoided. The output of cerebral mapping to acupuncture may be reflected in a normal physiological state. Moreover, to such a specific disorder, the psychological instruction could be implanted definitely and specifically.

Among the confounding factors in acupuncture studies, the most variable item is the psychological condition of subjects toward acupuncture therapy. Expectancy and belief have been proven to modulate the neuronal substrates of pain treated by acupuncture. [12] In this study, we introduced a distinct expectancy factor, not for its therapeutic efficacy for myopia but for its confounding effect in neuronal imaging of acupuncture stimulation. In all three contrasting conditions aimed to elicit cerebral responses to expectancy, the ipsilateral MFG, contralateral OFC, contralateral secondary sensory cortex (SII) and contralateral cerebellum were activated. The contralateral DLPFC, temporal pole and hippocampi uncus were activated in EG vs. NE and Group A vs. Group B. Many studies have validated the medial frontal gyrus- and DLPFC-related expectancy, emotion and cognitive control. [11], [22], [23] The temporal pole and hippocampi uncus are classical areas related to emotion. The activation of MFG is pronounced, so we proposed that the activity of the medial gyrus is involved in the modulation of acupuncture expectation. The function of the cerebellum has been confirmed to be not just limited to motor control but to play an important role in cognition and emotion including expectation. [23], [24] Given the complicated function of the cerebellum and our experimental design, we argued that the activation of the cerebellum in this study was related to expectancy.

### Conclusions

After filtering out confounding non-acupuncture factors, stimulation at LR3 was shown to activate some areas of the visual cortex. Such a result provided more evidence regarding the credibility of acupuncture therapy. On the other hand, cerebral responses to some non-acupuncture factors were complex and occurred in multiple areas, which may overlap with the neuronal responses to acupuncture. More precise experimental designs are needed to eliminate sources of bias in acupuncture controlled trials and to obtain sound results.

## References

[1] Fang B, Hayes JC (1999) Functional MRI explores mysteries of acupuncture. Diagn Imaging (San Franc) 21: 19–21. 10539699

[2] Hui KK, Liu J, Makris N, Gollub RL, Chen AJ, et al (2000) Acupuncture modulates the limbic system and subcortical gray structures of the human brain: evidence from fMRI studies in normal subjects. Hum Brain Mapp 9: 13–25. 10643726 10.1002/(SICI)1097-0193(2000)9:1<13::AID-HBM2>3.0.CO;2-F PMC6871878

[3] Wu MT, Sheen JM, Chuang KH, Yang P, Chin SL, et al (2002) Neuronal specificity of acupuncture response: a fMRI study with electroacupuncture. Neuroimage 16: 1028–1037. 12202090 10.1006/nimg.2002.1145

[4] Yan B, Li K, Xu J, Wang W, Li K, et al (2005) Acupoint-specific fMRI patterns in human brain. Neurosci Lett 383: 236–240. 15876491 10.1016/j.neulet.2005.04.021

[5] Yoo SS, Teh EK, Blinder RA, Jolesz FA (2004) Modulation of cerebellar activities by acupuncture stimulation: evidence from fMRI study. Neuroimage 22: 932–940. 15193624 10.1016/j.neuroimage.2004.02.017

[6] Vincent C, Lewith G (1995) Placebo controls for acupuncture studies. J R Soc Med 88: 199–202. 7745565 PMC1295163

[7] Lewith GT, Machin D (1983) On the evaluation of the clinical effects of acupuncture. Pain 16: 111–127. 6348651 10.1016/0304-3959(83)90202-6

[8] Dundee JW, McMillan CM (1992) P6 acupressure and postoperative vomiting. Br J Anaesth 68: 225–226. 1540472 10.1093/bja/68.2.225-a

[9] Kong J, Ma L, Gollub RL, Wei J, Yang X, et al (2002) A pilot study of functional magnetic resonance imaging of the brain during manual and electroacupuncture stimulation of acupuncture point (LI-4 Hegu) in normal subjects reveals differential brain activation between methods. J Altern Complement Med 8: 411–419. 12230901 10.1089/107555302760253603

[10] Wu MT, Hsieh JC, Xiong J, Yang CF, Pan HB, et al (1999) Central nervous pathway for acupuncture stimulation: localization of processing with functional MR imaging of the brain–preliminary experience. Radiology 212: 133–141. 10405732 10.1148/radiology.212.1.r99jl04133

[11] Wager TD, Rilling JK, Smith EE, Sokolik A, Casey KL, et al (2004) Placebo-induced changes in FMRI in the anticipation and experience of pain. Science 303: 1162–1167. 14976306 10.1126/science.1093065

[12] Pariente J, White P, Frackowiak RS, Lewith G (2005) Expectancy and belief modulate the neuronal substrates of pain treated by acupuncture. Neuroimage 25: 1161–1167. 15850733 10.1016/j.neuroimage.2005.01.016

[13] Vincent CA, Richardson PH (1986) The evaluation of therapeutic acupuncture: concepts and methods. Pain 24: 1–13. 3513094 10.1016/0304-3959(86)90022-9

[14] Park H, Park J, Lee H, Lee H (2002) Does Deqi (needle sensation) exist? Am J Chin Med 30: 45–50. 12067096 10.1142/S0192415X02000053

[15] Kim MS, Seo KM, Nam TC (2005) Effect of acupuncture on intraocular pressure in normal dogs. J Vet Med Sci 67: 1281–1282. 16397392 10.1292/jvms.67.1281

[16] Uhrig S, Hummelsberger J, Brinkhaus B (2003) Standardized acupuncture therapy in patients with ocular hypertension or glaucoma–results of a prospective observation study. Forsch Komplementarmed Klass Naturheilkd 10: 256–261. 14605482 10.1159/000074780

[17] Ghose GM, Yang T, Maunsell JH (2002) Physiological correlates of perceptual learning in monkey V1 and V2. J Neurophysiol 87: 1867–1888. 11929908 10.1152/jn.00690.2001

[18] Zhang WT, Jin Z, Huang J, Zhang L, Zeng YW, et al (2003) Modulation of cold pain in human brain by electric acupoint stimulation: evidence from fMRI. Neuroreport 14: 1591–1596. 14502082 10.1097/00001756-200308260-00010

[19] Price DD (2000) Psychological and neural mechanisms of the affective dimension of pain. Science 288: 1769–1772. 10846154 10.1126/science.288.5472.1769

[20] Craig AD (2003) Interoception: the sense of the physiological condition of the body. Curr Opin Neurobiol 13: 500–505. 12965300 10.1016/s0959-4388(03)00090-4

[21] Streitberger K, Kleinhenz J (1998) Introducing a placebo needle into acupuncture research. Lancet 352: 364–365. 9717924 10.1016/S0140-6736(97)10471-8

[22] Ridderinkhof KR, van den Wildenberg WP, Segalowitz SJ, Carter CS (2004) Neurocognitive mechanisms of cognitive control: the role of prefrontal cortex in action selection, response inhibition, performance monitoring, and reward-based learning. Brain Cogn 56: 129–140. 15518930 10.1016/j.bandc.2004.09.016

[23] Ueda K, Okamoto Y, Okada G, Yamashita H, Hori T, et al (2003) Brain activity during expectancy of emotional stimuli: an fMRI study. Neuroreport 14: 51–55. 12544830 10.1097/00001756-200301200-00010

[24] Schmahmann JD, Pandya DN (1997) The cerebrocerebellar system. Int Rev Neurobiol 41: 31–60. 9378595 10.1016/s0074-7742(08)60346-3

